# Risk Assessment of Delayed Graft Function in Pediatric Kidney Transplantation – a CERTAIN Research Network Analysis

**DOI:** 10.3389/ti.2026.14640

**Published:** 2026-03-19

**Authors:** Christian Patry, Miriam Boßung, Manuel Feißt, Kai Krupka, Britta Höcker, Lars Pape, Nele Kanzelmeyer, Lutz T. Weber, Jun Oh, Atif Awan, Thomas Simon, Licia Peruzzi, Ali Duzova, Jon Jin Kim, Mohan Shenoy, Claus Peter Schmitt, Alexander Fichtner, Burkhard Tönshoff

**Affiliations:** 1 Department of Pediatrics I, Medical Faculty, Heidelberg University, University Children’s Hospital Heidelberg, Heidelberg, Germany; 2 Institute of Medical Biometry, University of Heidelberg, Heidelberg, Germany; 3 Clinic for Paediatrics III, Essen University Hospital, Essen, Germany; 4 Department of Pediatric Kidney, Liver, Metabolic and Neurological Diseases, Hannover Medical School, Hannover, Germany; 5 Pediatric Nephrology, Children’s and Adolescents’ Hospital, University Hospital Cologne, Medical Faculty University of Cologne, Cologne, Germany; 6 Pediatric Nephrology, University Hospital Hamburg, Hamburg, Germany; 7 Department of National Paediatric Renal Transplantation, Children’s Health Ireland at Temple Street, Dublin, Ireland; 8 Pediatric Nephrology, Toulouse University Hospital, Toulouse, France; 9 Pediatric Nephrology Unit, Regina Margherita Children’s Hospital, AOU Città della Salute e della Scienza di Torino, Torino, Italy; 10 Division of Pediatric Nephrology, Hacettepe University Faculty of Medicine, Ankara, Türkiye; 11 Department of Paediatric Nephrology, Nottingham University Hospital, Nottingham, United Kingdom; 12 Paediatric Nephrology, Royal Manchester Children’s Hospital, Manchester, United Kingdom

**Keywords:** delayed graft function, risk assessment model, pediatric kidney transplantation, post-transplant complications, ischemia-reperfusion injury

## Abstract

Delayed graft function (DGF) in pediatric kidney transplantation is a serious complication with negative impact on graft survival. Currently, there are no reliable methods available to assess the risk of DGF in children. We performed a retrospective analysis of data from the Cooperative European Paediatric Renal Transplant Initiative (CERTAIN) registry to develop a DGF risk assessment model for pediatric kidney transplantation, based on parameters available within the first 24 h post-transplant. The model was developed by forward selection and logistic regression. This study included n = 694 patients. The overall rate of DGF was 8.5%. The following key parameters were selected for the DGF risk assessment model: (i) occurrence of post-transplant surgical complications, (ii) immediate graft urine production, (iii) rate of change in recipient’s serum creatinine, (iv) initial calcineurin inhibitor therapy. The significance of these parameters was confirmed by calculating adjusted odds ratios. In the training cohort and the internal validation cohort the ROC-AUCs were 0.9043 and 0.878. This multivariable model based on early post-transplant parameters can predict the occurrence of DGF in pediatric kidney transplant recipients with high accuracy and may facilitate future interventional trials of targeted pharmacological strategies against ischemia-reperfusion injury in this population.

## Graphical Abstract

**Figure:**
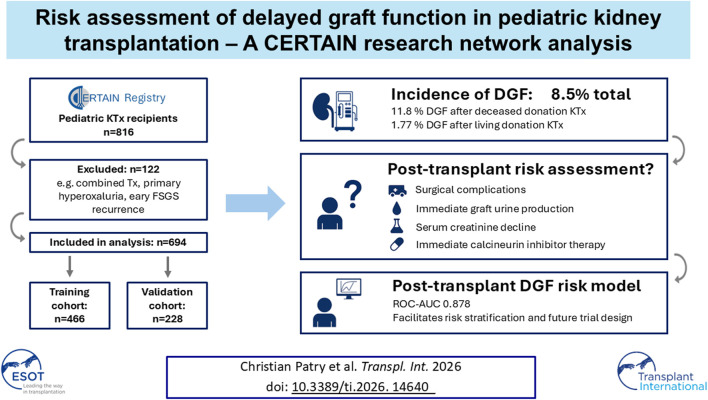

## Introduction

Delayed graft function (DGF) in newly transplanted kidneys is currently defined as the need for dialysis within the first 7 days after transplantation [1]. In children, the reported incidence of DGF after deceased donor transplantation ranges from 7.5% to 19.7%, with a decreasing incidence in recent years [2]. According to data from UK and US databases, it is lower in living-related transplants (4%) [2, 3]. DGF in children is associated with adverse long-term outcomes, such as higher rates of rejection and decreased graft survival [4, 5].

DGF is primarily caused by ischemia-reperfusion injury, which involves microvascular inflammation, the production of reactive oxygen species and cell death [6]. Currently, both preclinical and clinical research is exploring potential therapeutic targets to intervene early in these pathways [7, 8]. Since DGF is a risk factor for graft dysfunction in both short- and long-term follow-up [4], children with a high likelihood of experiencing ischemia-reperfusion injury and subsequent occurrence of DGF may benefit from such therapeutic strategies for ischemia-reperfusion injury in the first days after transplantation surgery. Methods to identify this respective high-risk pediatric subpopulation early in the initial clinical course after transplantation would facilitate future interventional clinical trials with targeted treatment options and could also provide additional guidance for immediate patient-specific management post-transplant. A limited number of studies in adults have examined factors associated with ischemia-reperfusion injury for their potential to aid in the assessment of the likelihood of the occurrence of DGF, such as delayed graft urine production [9–11]. However, factors associated with DGF in adult kidney transplant recipients cannot be uncritically extrapolated to children, as important variables unique to the pediatric population may be overlooked. In pediatric kidney transplantation, there is a complete lack of statistical models based on post-transplant parameters that could rapidly identify those children who are likely to have experienced ischemia-reperfusion injury and therefore are at increased risk of developing DGF.

Therefore, we designed this study based on data from the Cooperative European Paediatric Renal Transplant Initiative (CERTAIN) registry [12–14]. We had two aims: First, we wanted to investigate peri- and post-transplant parameters that might be associated with graft ischemia-reperfusion injury and thus might help predict the occurrence of DGF. Second, we wanted to develop a predictive model for DGF in children based on these peri- and/or post-transplant parameters.

## Materials and Methods

### Patients and Data Collection

This retrospective, multicenter, longitudinal cohort study included pediatric kidney transplant recipients enrolled in the CERTAIN registry. The CERTAIN registry collects detailed longitudinal clinical and laboratory data and applies rigorous validity checking procedures^1^. Participation in the CERTAIN registry was approved by the ethics committee at each center. Informed consent was obtained from the parents or legal guardians prior to enrollment, with assent from patients when appropriate for their age. This general ethics approval for participation in the registry fully covers the use of the collected data for this registry-based study. This general ethics approval for the registry fully covers the use of the collected data for the present study. The time points of data collection, and the corresponding time intervals were as follows: baseline (pre-transplant), at months 1, 3, 6, 9, 12 and every 6 months thereafter up to 5 years post-transplant. One graft per patient was analyzed. A more detailed description of the CERTAIN registry with respect to data quality and completeness can be found in the Supplementary Appendix.

**FIGURE 1:**
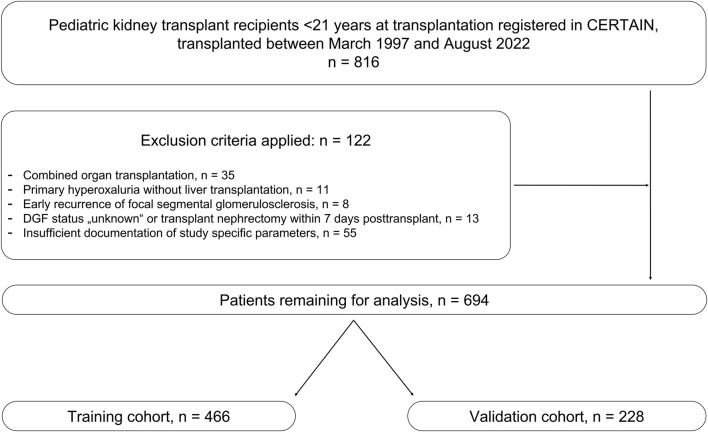
Study flowchart for the development of the post-transplant pediatric DGF risk assessment model. Post-transplant data were evaluated specifically for this model in n = 816 patients. After application of exclusion criteria, n = 694 patients remained, which were further divided 2:1 into a training cohort and a validation cohort. DGF, delayed graft function.

Patients were eligible for the study if they received a kidney transplant before the age of 21 years. Exclusion criteria were early graft nephrectomy within the first week after transplantation, primary hyperoxaluria as primary kidney disease, combined transplants, or early recurrence of focal segmental glomerulosclerosis post-transplant (Figure 1).

### Study Design

Data from both deceased and living donor transplants in children transplanted between March 1997 and August 2022 were included. The study was designed, analyzed and reported according to the Strengthening the Reporting of Observational Studies in Epidemiology (STROBE) guidelines^2^. Patients who experienced DGF are referred to as the “DGF group,” and the others as the “no-DGF group”. We focused on risk factors occurring within the first 24 h post-transplant to allow early identification of at-risk patients who might benefit from pharmacologic intervention. The following post-transplant parameters were selected based on evidence from the available literature [9–11] and/or clinical plausibility to be evaluated in recipients during the first 24 h after kidney transplantation surgery: post-transplant complications requiring re-operation, rate of change in recipient’s serum creatinine, systolic and diastolic blood pressure, central venous blood pressure, need for vasopressor therapy, urine production, furosemide therapy, and start of calcineurin inhibitor therapy within the first 24 h after kidney transplantation (Table 1). Parameters not documented in the CERTAIN registry minimum data set were documented in a study-specific electronic case report form designed specifically for this project. In addition, we assessed perioperative parameters specific to this study, namely, “type of donor kidney (left/right)”, “type of arterial vessel anastomosis (aorta/iliac)”, and “type of venous vessel anastomosis (cava/iliac externa/iliac communis/other)” (Table 1). Finally, we developed a DGF risk assessment model based on the analyzed parameters. We report this model according to the guidelines of the Transparent Reporting of a Multivariable Prediction Model for Individual Prognosis or Diagnosis (TRIPOD) statement (see the Supplementary Appendix) [15]. In a subcohort of 680 patients (n = 56 in the DGF group, n = 624 in the no DGF group), we investigated whether pre-transplant factors that might influence the occurrence of DGF post-transplant could further enhance the predictive accuracy of the developed prediction model (Supplementary Figure 1). The following pre-transplant parameters were included in this extended analysis: pre-transplant dialysis mode, donor type, donor sex, recipient age, duration of cold ischemia time, donor hemodynamic instability (see definition below) and number of HLA-DR mismatches (Supplementary Table 1). No patient was treated following a steroid avoidance protocol.

**TABLE 1:** Parameters examined in the analysis of peri- and post-transplant risk factors for DGF.

Parameters of interest	DGF, n = 59	no DGF, n = 635	*P* value
Recipient			
Age at kidney transplantation [years], mean ± SD	10.5 ± 5.9	9.8 ± 5.3	0.365
Type of donor kidney, n (%)	​	​	0.665
Left donor kidney	27 (47)	311 (50)	​
Right donor kidney	31 (51)	317 (50)	​
Missing values	1	7	​
Arterial vessel anastomosis, n (%)	​	​	0.447
Aorta	21 (37)	261 (42)	​
Iliac vessels	36 (63)	360 (58)	​
Missing values	2	14	​
Venous vessel anastomosis, n (%)	​	​	0.460
Vena cava	27 (47)	354 (57)	​
Vena iliac externa	11 (19)	110 (18)	​
Vena iliac communis	13 (23)	98 (16)	​
Other venous vessel	6 (11)	58 (9)	​
Missing values	2	15	​
Surgical complications post-transplant	​	​	<0.001
No	51 (88)	616 (99)	​
Yes	7 (12)	7 (1)	​
Missing value	1	12	​
Rate of change in recipient’s serum creatinine, mean ± SD (DGF, n = 59; no-DGF, n = 632)	−0.008 ± 0.025	−0.064 ± 0.092	<0.001
Systolic blood pressure, mean ± SD [mmHg]			
Mean pressure between 0 and 12 h (DGF, n = 54; no-DGF, n = 518)	123 ± 18	121 ± 18	0.342
Mean pressure between 12 and 24 h (DGF, n = 52; no-DGF, n = 498)	126 ± 17	123 ± 17	0.336
Minimum pressure between 0 and 12 h (DGF, n = 54; no-DGF, n = 518)	113 ± 21	112 ± 19	0.602
Minimum pressure between 12 and 24 h (DGF, n = 51; no-DGF, n = 487)	121 ± 18	118 ± 18	0.310
Maximum pressure between 0 and 12 h (DGF, n = 54; no-DGF, n = 518)	113 ± 18	130 ± 20	0.290
Maximum pressure between 12 and 24 h (DGF, n = 51; no-DGF, n = 487)	132 ± 19	128 ± 18	0.137
Diastolic blood pressure, mean ± SD [mmHg]			
Mean pressure between 0 and 12 h (DGF, n = 54; no-DGF, n = 518)	68 ± 18	66 ± 15	0.563
Mean pressure between 12 and 24 h (DGF, n = 52; no-DGF, n = 498)	72 ± 16	70 ± 14	0.397
Minimum pressure between 0 and 12 h (DGF, n = 54; no-DGF, n = 518)	61 ± 20	60 ± 16	0.599
Minimum pressure between 12 and 24 h (DGF, n = 51; no-DGF, n = 487)	68 ± 17	65 ± 15	0.252
Maximum pressure between 0 and 12 h (DGF, n = 54; no-DGF, n = 518)	74 ± 18	72 ± 16	0.554
Maximum pressure between 12 and 24 h (DGF, n = 51; no-DGF, n = 487)	77 ± 16	75 ± 15	0.299
MAP, mean ± SD [mmHg]			
Mean pressure between 0 and 12 h (DGF, n = 50; no-DGF, n = 457)	85 ± 17	85 ± 15	0.821
Mean pressure between 12 and 24 h (DGF, n = 50; no-DGF, n = 465)	89 ± 15	88 ± 14	0.752
Minimum pressure between 0 and 12 h (DGF, n = 50; no-DGF, n = 457)	78 ± 18	77 ± 15	0.848
Minimum pressure between 12 and 24 h (DGF, n = 49; no-DGF, n = 451)	85 ± 16	84 ± 15	0.470
Maximum pressure between 0 and 12 h (DGF, n = 50; no-DGF, n = 457)	93 ± 17	93 ± 16	0.836
Maximum pressure between 12 and 24 h (DGF, n = 49; no-DGF, n = 451)	94 ± 15	92 ± 16	0.347
Central venous blood pressure, mean ± SD [mmHg]			
Mean pressure between 0 and 12 h (DGF, n = 40; no-DGF, n = 358)	6.7 ± 3.7	5.1 ± 3.6	0.010
Mean pressure between 12 and 24 h (DGF, n = 39; no-DGF, n = 326)	6.9 ± 3.4	5.7 ± 4.3	0.045
Minimum pressure between 0 and 12 h (DGF, n = 40; no-DGF, n = 358)	5.4 ± 3.9	3.9 ± 3.8	0.031
Minimum pressure between 12 and 24 h (DGF, n = 36; no-DGF, n = 300)	6.6 ± 3.8	4.9 ± 3.7	0.015
Maximum pressure between 0 and 12 h (DGF, n = 40; no-DGF, n = 358)	8.1 ± 3.9	6.3 ± 4.1	0.007
Maximum pressure between 12 and 24 h (DGF, n = 36; no-DGF, n = 300)	7.7 ± 3.4	7.1 ± 7.3	0.411
Vasopressor therapy, n (%)	​	​	<0.001
None	20 (35)	302 (56)	​
(Nor-)epinephrine	27 (47)	115 (21)	​
Dopamine	9 (16)	115 (21)	​
Other vasopressors	1 (2)	11 (2)	​
Missing values	2	92	​
Urine production, n (%)	​	​	<0.001
No	27 (69)	64 (17)	​
Yes	12 (31)	308 (83)	​
Missing values	20	263	​
Furosemide therapy, n (%)	​	​	0.182
No	8 (15)	122 (23)	​
Yes	45 (85)	406 (77)	​
Missing values	6	107	​
Initial calcineurin inhibitor therapy, n (%)^1^	​	​	0.293
No	3 (6)	16 (3)	​
Yes	50 (94)	520 (97)	​
Missing values	5	99	​

For binary and categorical variables, numbers and percentages are given. For continuous variables, mean and standard deviation are presented. A two-sided t-test was calculated for metric variables, chi-squared test was applied to compare non-metric variables. For all tests *P* < 0.05 was considered significant. ^1^Initial calcineurin inhibitor therapy: 85% tacrolimus, 15% cyclosporin.

MAP, mean arterial pressure.

### Definitions of Study-Specific Variables

The variable “complications after kidney transplantation requiring reoperation” included at least one of the following events: thrombosis (DGF group, n = 5; no DGF group, n = 2), bleeding (DGF group, n = 3; no DGF group, n = 4), or vascular stenosis (DGF group, n = 0; no DGF group, n = 2) within 24 h post-transplant. If any of these three complications occurred, the patient was classified as having a post-transplant complication. There were no other reasons for reoperation within the first 24 h post-transplant. “Immediate urine production” was defined as urine production by the graft, either during transplant surgery or in the first 24 h thereafter. Urine production was assessed in n = 552 patients (79.5%) by direct drainage through a catheter stenting the graft ureter (n = 507 patients, 79.8% in the DGF group and n = 45 patients, 76.3% in the no DGF group). Supplementary Table 2 shows the type of graft ureteral stent used. The variable “vasopressor therapy” had four categories, which were “none,” “(nor)epinephrine,” “dopamine,” and “other vasopressors.” If the patient received more than one vasopressor including (nor)epinephrine, they were classified as treated with (nor)epinephrine. If the patient received combination therapy that included dopamine but not (nor)epinephrine, the patient was classified as receiving dopamine. If a vasopressor other than (nor)epinephrine or dopamine was used, the patient was classified in the “other vasopressor” category. In n = 17 patients, the parameter: “other vasopressor” included dobutamine. The variable “rate of change in recipient’s serum creatinine” was mathematically modeled using Formula 1, assuming an exponential decay process:

$$\sqrt[{\left(\mathrm{(T)}\right)}]{1-\frac{{\text{recipient}}^{\prime }s\;\text{serum}\;\text{crea}\;\text{pretransplant}\;\left[\frac{\text{mg}}{\text{dl}}\right]-{\text{recipient}}^{\prime }s\text{ serum crea at timepoint }T\text{ posttransplant}\left[\frac{\text{mg}}{\text{dl}}\right]\;}{{\text{recipient}}^{\prime }s\text{ serum crea }\text{pretransplant}\left[\frac{\text{mg}}{\text{dl}}\right]}}-1$$ (1)

Definitions: T = time point of recipient’s serum creatinine measurement within the first 24 h after transplant surgery [hours]. The time point of graft reperfusion after transplantation surgery was defined as T = 0; the time point 24 h after reperfusion was defined as T = 24. Crea = creatinine.

In the subgroup analysis of pre- and post-transplant predictors of DGF the variable “donor hemodynamic instability” was defined as the presence of at least one of the following parameters: absence of spontaneous cardiac activity, occurrence of hypotensive episodes as documented in CERTAIN, treatment of the donor with norepinephrine and/or dopamine.

### Statistical Analyses

The risk assessment model was developed using multiple logistic regression based on forward selection of individual variables. Patient and graft characteristics were described by mean and standard deviation for continuous variables and relative and absolute frequencies for categorical variables. First, the peri- and post-transplant parameters assessed in this study were compared between DGF and no-DGF patients using univariable t-tests or chi-squared tests, as appropriate. Second, these parameters were analyzed using the variable selection process. To develop the risk assessment model, patients were randomly assigned to a training and a test cohort in a 2:1 ratio to ensure equal prevalence of the outcome „occurrence of DGF”. Multiple imputation was then performed on the training data. Imputation was based on the fully conditional specification method of Van Buuren et al. [16] and performed with the “mice” package (using 20 imputations). Forward selection was applied to the imputed data sets for logistic regression modeling. Forward selection based on 1000-fold subsampling was performed on 63.2% (1-1/e) of the cohort for each imputed data set. Variables selected for the final model had to be both selected in all imputed data sets for the full training cohort and also to be confirmed in the subsampling process. The final model was built by pooling the respective sub-models derived from the multiple imputation process. To control for interdependence of model parameters and to provide transparency following the guidelines of the TRIPOD statement [15], we calculated adjusted odds ratios for each parameter selected for the model. Internal validation of the final model was performed (i) only on complete cases with no missing data in the test cohort, (ii) on each imputed dataset (same procedure as for the training cohort), and (iii) on a combined multiple imputed data set, which was derived by averaging. Validation was assessed by the area under the receiver operating characteristic curve (ROC-AUC). Model calibration was assessed using calibration plots. All analyses were performed with R version >4.0.0.

## Results

### Patient and Transplant Characteristics

We screened n = 816 patients. After applying the exclusion criteria, n = 694 children remained for further analysis (Figure 1). The overall rate of DGF in the entire patient cohort was 59 out of 694 patients (8.5%); the rate of DGF after deceased donation was 11.8% (55 out of 468 patients), after living donation 1.77% (4 out of 226 patients) (*P* < 0.001). Table 2 shows the baseline characteristics of patients with and without DGF and the corresponding donor characteristics. Patients with DGF were more likely to be male, less likely to have at least one HLA-DR mismatch, and less likely to have undergone preemptive transplantation. 93.2% of patients with DGF had received a transplant from a deceased donor and 6.8% from a living donor. Cold ischemia time in patients with DGF (821 ± 360 min) was 34.6% longer (*P* < 0.001) than in patients without DGF (610 ± 424 min). Supplementary Table 3 shows the timepoint of initiation of post-transplant dialysis within the first 7 days after transplant surgery in the DGF cohort.

**TABLE 2:** Comparison of baseline recipient and donor characteristics in patients with and without DGF.

Characteristics	DGF n = 59	no DGF n = 635	*P* value
Recipient			
Age [years], mean ± SD	10.5 ± 5.9	9.8 ± 5.3	0.316
Male sex, n (%)	43 (72.0)	378 (59.5)	0.045
Height [cm], mean ± SD	130.1 ± 33.5	126.2 ± 31.4	0.363
Weight [kg], mean ± SD	33.8 ± 20.2	30.8 ± 17.7	0.228
BMI [kg/m^2^], mean ± SD	17.9 ± 3.4	17.6 ± 3.2	0.478
Number of previous kidney transplantations, n > 0 (%)	6 (10.2)	55 (8.6)	0.302
Patients receiving anti-thymocyte therapy, n (%)	1 (1.69)	10 (1.57)	0.943
At least one HLA mismatch			
HLA-A	34 (57.7)	424 (66.8)	0.258
HLA-B	45 (76.2)	501 (78.9)	0.567
HLA-DR	38 (64.4)	462 (72.7)	0.035
Primary kidney disease, n (%)	​	​	0.2717
Renal hypoplasia/dysplasia	15 (25.4)	174 (27.4)	​
Glomerular disease	18 (30.5)	148 (23.3)	​
Obstructive uropathy/vesicoureteral reflux	6 (10.2)	93 (14.6)	​
Polycystic kidney disease	9 (15.3)	89 (14.0)	​
Not specified/unknown	1 (1.7)	71 (11.2)	​
Tubular disorder	4 (6.8)	21 (3.3)	​
Systemic disorders including vasculitis	2 (3.4)	13 (2.0)	​
Kidney tumor	2 (3.4)	13 (2.0)	​
Other	2 (3.4)	13 (2.0)	​
Dialysis prior to KTx, n (%)	​	​	<0.001
Preemptive KTx	2 (3.4)	181 (28.5)	​
Hemodialysis	26 (44.1)	183 (28.8)	​
Peritoneal dialysis	31 (52.2)	267 (42.1)	​
Unknown	0 (0.0)	4 (0.6)	​
Donor			
Age [years], mean ± SD	31.0 ± 16.6	30.0 ± 17.6	0.683
Type of donor			
Deceased donor after brain death, n (%)	43 (72.9)	305 (48.0)	<0.001
Deceased donor after cardiac death, n (%)	2 (3.39)	10 (1.57)	<0.001
Deceased donor, unknown reason, n (%)	10 (16.9)	98 (15.4)	<0.001
Living donor, n (%)	4 (6.8)	222 (35.0)	<0.001
Cold ischemia time [min], mean ± SD	821 ± 359.8	610 ± 424	<0.001

For binary and categorical parameters, numbers and percentages are presented. For continuous variables, mean and standard deviation are presented.

A two-tailed t-test was calculated for metric variables. Chi-squared test was used to compare non-metric variables. For all tests, p < 0.05 was considered significant.

BMI, body mass index; KTx, kidney transplantation; HLA, human leukocyte antigen.

### Peri- and Post-Transplant Risk Factors for DGF

Table 1 shows the analyzed peri- and post-transplant risk factors for DGF. Post-transplant complications requiring reoperation were significantly more frequent in the DGF group (12%) than in the no-DGF group (1%, *P* < 0.001). The rate of decline in recipient’s serum creatinine (calculated by Formula 1, see above) during the first 24 h post-transplant was significantly slower in the DGF group (−0.008 ± 0.025 = 0.8% ± 2.5% decline per hour) than in the no-DGF group (−0.064 ± 0.092 = 6.4% ± 9.2% decline per hour; *P* < 0.001). The frequency of vasopressor use was significantly different between the DGF and the no-DGF groups. Norepinephrine and epinephrine were used more frequently in the DGF group (47%) than in the no-DGF group (21%, *P* < 0.001), whereas dopamine was used more frequently in the no-DGF group (21%) than in the DGF group (16%, *P* < 0.001). Immediate urine production was significantly more frequent in the no-DGF group (83%) than in the DGF group (31%, *P* < 0.001). Several measures of central venous pressure were significantly higher in the DGF group than in the no-DGF group, whereas the measures systolic blood pressure, diastolic blood pressure, and mean arterial pressure (MAP) were comparable between groups (Table 1).

### Parameter Selection and Performance of the Model

The total patient population was divided 2:1 into a training cohort (n = 466 patients) and a validation cohort (n = 228) (Figure 1). The DGF risk assessment model was trained in the training cohort using logistic regression analysis on the parameters listed in Table 1. The following combination of four key post-transplant parameters was selected for the DGF risk assessment model by the forward selection process: (i) occurrence of post-transplant surgical complications, (ii) immediate urine production, (iii) rate of change in recipient’s serum creatinine, (iv) initial calcineurin inhibitor therapy (Table 3). In addition, the significance of the association of each of these four post-transplant parameters with the occurrence of DGF was confirmed by calculating adjusted odds ratios (Table 3). Post-transplant surgical complications and slower rate of decline in recipient’s serum creatinine were associated with increased odds of DGF, whereas immediate urine production and initial calcineurin inhibitor were associated with decreased odds of DGF.

**TABLE 3:** Parameters selected for the DGF risk assessment model.

Parameters	Regression coefficient	Odds ratio [95% CI]	*P* value
*Intercept*	*2.316*	10.132 [1.899–50.364]	0.008
Surgical complications post-transplant			
No	*Reference*	-	-
Yes	1.694	5.443 [1.236–26.208]	0.026
Immediate urine production			
No	*Reference*	-	-
Yes	−2.408	0.090 [0.033–0.211]	<0.001
Initial calcineurin inhibitor therapy			
No	Reference	-	-
Yes	−2.978	0.051 [0.011–0.256]	<0.001
Rate of change in recipient’s serum creatinine	0.233	1.262 [1.107–1.467]	<0.001

The reference category describes the variable characteristic to which the risk calculation of the other parameters refers. The reference category was randomly selected.

**FIGURE 2:**
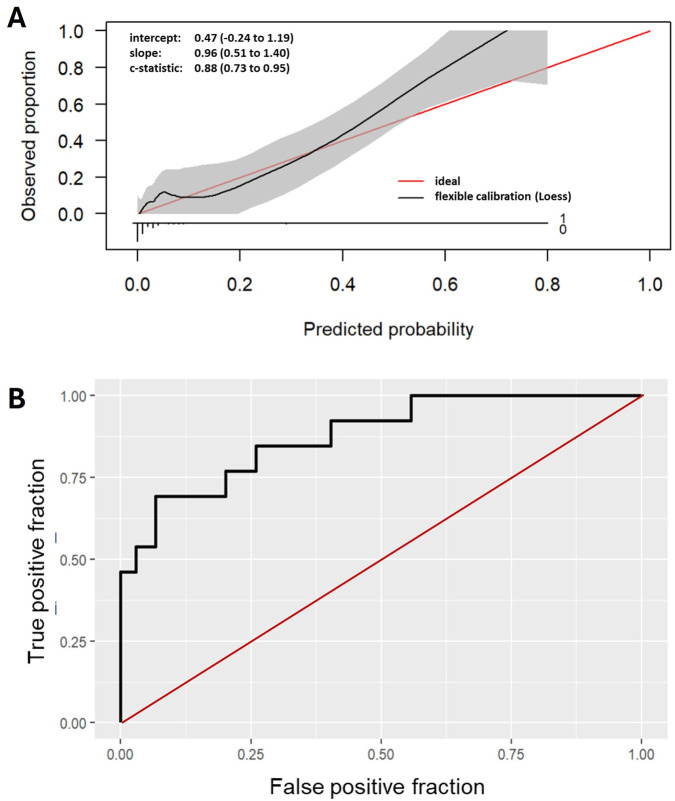
**(A)** shows the calibration plot of the pediatric DGF risk assessment model. **(B)** shows the ROC-AUC curve for the model, illustrating the discriminative performance. ROC-AUC, area under the receiver operating characteristic curve.

In the training cohort, the corresponding ROC-AUC of the final model was 0.9043. In a second step, the imputation procedures generated several validation cohorts with imputed data for parameters with missing data. The mean ROC-AUC of the model used on these imputed datasets was 0.8765. After merging the imputed datasets, an imputed validation cohort was generated with a ROC-AUC of 0.9104 in the model. In comparison, the ROC-AUC of the validation cohort without imputed data was 0.878 (Table 4; Figure 2). The corresponding risk of developing DGF based on post-transplant parameters can be calculated with the model using the following formula (Formula 2) together with the coefficients written below and shown in Table 3.

$$\text{Linear Score}=2.316+\text{Variable }A+\text{Variable }B+\text{Variable }C+\left(0.233\times Variable\;D\right)$$

$$P\;\left(\text{DGF}\right)=\frac{1}{1+e^{-L\text{inear}\;\text{Score}\;}}$$ (2)

**TABLE 4:** ROC-AUCs of the DGF risk assessment model in different patient cohorts.

Type of cohort	ROC-AUC
ROC-AUC on training cohort	0.9043
ROC-AUC on validation cohort with complete cases	0.878
Mean ROC-AUC of imputation validation datasets	0.8765
ROC-AUC on merged imputed validation cohort	0.9104

ROC-AUC, area under the receiver operating characteristic curve.

There were no missing data in the selected predictors.

Definitions:

- P (DGF) = probability of developing DGF predicted by the DGF risk assessment model (range: 0–1)
- Variable A: post-transplant surgical complications occurred = 1.694; post-transplant surgical complications did not occur = 0
- Variable B: immediate urine production = −2.408; no immediate urine production = 0
- Variable C: initial calcineurin inhibitor therapy = −2.978; no initial calcineurin inhibitor therapy = 0
- Variable D: rate of change in recipient’s serum creatinine (see Formula 1)

Abbreviations: DGF, delayed graft function. An online calculator based on Formula 2 will be available on the CERTAIN website at the time of publication of this article.

### Calibration of Final Risk Assessment Model

The calibration of this final pediatric DGF risk assessment model was evaluated using calibration plots. The corresponding calibration plot is shown in Figure 2. The model showed the following performance in terms of calibration: Intercept: 0.47; slope: 0.96. Due to the low prevalence of DGF in the study cohort, there are some deviations at very low and high predicted probabilities. Additionally, the confidence intervals for the intercept (−0.24 – 1.19) and slope (0.51–1.40) are relatively broad. Although the C-statistic of 0.88 indicates that the model has high discriminative power, at very high predicted probabilities (>0.8), the model appears to slightly overestimate the probability of the event (Figure 2).

### Extended Analysis of Combined Predictors for DGF

In the extended analysis of combined pre- and post-transplant risk factors for DGF, we used the same statistical methods and procedures as used in the main analysis (described above). We were able to demonstrate that the inclusion of additional pre-transplant parameters did not improve the predictive performance of the primarily developed prediction model based on post-transplant parameters alone, as identified in the main analysis (see above). Supplementary Table 1 shows all pre- and post-transplant parameters examined in this extended analysis. For the combined model, only the number of HLA-DR mismatches was additionally selected by the forward selection procedure (Supplementary Table 4). The adjusted odds ratio for the selected pre-transplant parameter “HLA-DR mismatches” was not statistically significant (Supplementary Table 4). The AUC (0.8782 in the validation cohort without imputed data) of this combined pre and post-transplant model was comparable to the AUC (0.878 in the validation cohort without imputed data) of the developed post-transplant prediction model. Supplementary Figure 2 shows the calibration plot of the combined pre -and post-transplant pediatric DGF risk prediction model.

### Sensitivity Analysis After Exclusion of the Variable “Initial Calcineurin Inhibitor Therapy”

Sensitivity analysis after exclusion of the variable ‘initial calcineurin inhibitor therapy: To assess the impact of the predictor ‘start of calcineurin inhibitor therapy within 24 h post-transplant’ on model performance, we repeated the forward selection procedure as in the main analysis. Then, we excluded the CNI variable post-selection and refitted the model. The three remaining predictors were highly significant (p < 0.001 for each parameter) and had regression coefficients comparable to those in the main model (surgical complications: 1.574 vs. 1.694; immediate urine production: −2.049 vs. −2.408; rate of change in serum creatinine: 0.233 vs. 0.233). The corresponding adjusted odds ratios were also comparable (surgical complications: 4.827 [95% CI: 1.147–21.35] vs. 5.443 [1.236–26.21]; immediate urine production: 0.128 [0.055–0.277] vs. 0.09 [0.033–0.211]; rate of change in serum creatinine: 1.263 [1.112–1.462] vs. 1.262 [1.107–1.467]; Supplementary Table 5). The discriminative performance of this three-variable model remained good, closely resembling that of the four-variable model (ROC-AUCs: 0.882 vs. 0.904 in the training cohort; 0.891 vs. 0.878 in the validation cohort with complete cases; a mean of 0.885 vs. 0.877 across imputed validation datasets; 0.917 vs. 0.910 in the merged imputed validation cohort; Supplementary Table 6.

## Discussion

This study is the first to develop a multivariable prediction model based on early (within the first 24 h after kidney transplant surgery) post-transplant parameters to assess the likelihood of DGF in pediatric recipients. This model can predict the occurrence of DGF in children with high accuracy, independent of pre-transplant risk factors. After external validation, this model can be used in two different clinical settings: First, the model can identify children at high risk for DGF; thus, it can serve as a valuable tool to guide patient-specific clinical decision-making after transplantation and to tailor management strategies accordingly. Second, the reliable identification of pediatric patients at high risk for DGF is important for immediate post-transplant clinical decision making and future interventional trials of targeted pharmacological strategies against ischemia-reperfusion injury. Ischemia-reperfusion injury resulting in DGF after kidney transplantation is a currently unmet clinical need. Several approaches to targeting ischemia-reperfusion injury pathways in the context of kidney transplantation are currently being evaluated in preclinical or clinical trials, including inhibition of complement pathways, modulation of complement regulatory proteins, or interference with the hepatocyte growth factor pathway [6, 17, 18]. Thus, this model may facilitate the conduct of such trials in children in the future.

We found that several early post-transplant parameters were significantly associated with the development of DGF. One of these factors was hemodynamic instability as indicated by the need for circulatory support with inotropes such as epinephrine or norepinephrine in the recipient. It is likely that hemodynamic instability in the recipient leads to hypoperfusion of the renal allograft. In addition, the use of inotropic agents such as norepinephrine may itself impair graft perfusion mediated by pharmacologically induced arteriolar constriction [19].

The DGF risk assessment model was trained in the training cohort using logistic regression analysis, selecting the following four key post-transplant parameters: (i) occurrence of post-transplant surgical complications, (ii) immediate urine production, (iii) initial calcineurin inhibitor therapy, (iv) rate of change in recipient’s serum creatinine. The significance of the association of each of these four parameters with the occurrence of DGF was confirmed by calculating adjusted odds ratios. The occurrence of postoperative complications requiring reoperation on the first day after transplantation had the highest odds ratio, indicating a 5.4-fold increase in the odds of DGF. A slower rate of decline in the recipient’s serum creatinine level on the first day post-transplant was associated with a 26% increase in the odds of DGF, while urine production immediately post-transplant was associated with a 90% decrease in the odds of DGF. Both parameters can be interpreted as markers of ischemia-reperfusion injury-mediated graft dysfunction. Initial immunosuppressive therapy with calcineurin inhibitors had an adjusted odds ratio of 0.051, indicating a 95% reduction in the odds of DGF. However, only 3 patients (6%) in the DGF cohort did not receive a calcineurin inhibitor compared to 16 patients (3%) in the no-DGF cohort (Table 2). Because of these small numbers, this finding should be interpreted with caution. In addition, children identified by treating physicians as being at high risk for DGF may have initially received a CNI-free immunosuppressive regimen. This may have confounded the observed association between CNI use and reduced risk of developing DGF. To further assess the impact of the immediate initiation of CNI treatment after KTx on overall model performance, we performed a sensitivity analysis that excluded the CNI variable after forward selection. This analysis showed that initial CNI use did not substantially impact the strength or direction of the associations between the other predictors and DGF. This finding supports the internal validity of the final pediatric post-transplant DGF risk assessment model. Accordingly, the model should not be interpreted as evidence of a protective effect of CNI, but rather as a tool for DGF risk stratification in children in the early post-transplant setting.

In a dedicated extended analysis, we evaluated established or putative pre-transplant risk factors for DGF development such as cold ischemia time and donor type [20] for their potential to improve the predictive accuracy of the post-transplant model developed in this study. None of the additional pre-transplant parameters improved the performance of the post-transplant prediction model. Among the pre-transplant factors analyzed, only the number of HLA-DR mismatches was included in the combined model using the forward selection process. However, this combined prediction model did not outperform the post-transplant model, as evidenced by a nearly identical AUC in the internal validation. Furthermore, the inclusion of HLA-DR mismatches in the combined prediction model, as suggested by forward selection, is unlikely to provide meaningful clinical benefit given the lack of statistical significance in an adjusted odds ratio analysis performed in parallel. Therefore, the results of this extended analysis of combined risk factors for DGF suggest that the investigated post-transplant parameters alone are sufficient for accurate prediction of DGF in pediatric kidney transplantation within 24 h after transplantation surgery.

The post-transplant DGF risk assessment model developed in this study demonstrated robust discrimination performance, but limitations regarding calibration, accuracy, generalizability, and clinical applicability must be considered. First, our study is retrospective and therefore subject to the biases inherent to patient registries. Variability in donor and recipient characteristics, transplant practices, or local protocols may limit the external validity of prediction models. Therefore, even if this DGF risk assessment model performs well in the internal validation process, a generalizability of its use will require an additional external validation step based on diverse pediatric transplant populations. Second, it is important to note that the predictive accuracy of this DGF risk assessment model is limited to the variables included in the analysis itself. Factors not analyzed in this study may also contribute to the development of DGF. However, we were unable to examine the method of organ procurement in living donors because the number of DGF events in the subset of living donors in this patient cohort was too small for a meaningful statistical analysis. In addition, the landscape of pediatric kidney transplantation continues to evolve, which may introduce other new potential variables for the development of DGF in the future. Potential changes in post-transplant treatment regimens in the future must be considered in a DGF risk prediction model to keep it current and ensure its continued applicability. Third, the absolute number of patients with DGF in this study cohort was relatively small. To avoid an overly optimistic estimation of the predictive accuracy of the DGF risk assessment model developed in this study, we performed a calibration analysis. Despite the low prevalence of DGF in our cohort, the analysis revealed a general alignment between the predicted and observed frequencies of DGF.

Fourth, the forward selection process applied in our study optimizes the model’s predictive performance but does not evaluate individual parameters independently for causality. The observed associations between the selected variables do not reflect isolated causal effects of single parameters, but their combined predictive contribution to the DGF risk in our study population. This is especially relevant regarding the observed negative association between early CNI use and DGF risk. The model should not be used to determine whether CNI therapy can be initiated or should be withheld. The model captures patient-specific factors present on day 1 post-transplant, including already taken clinical decisions such as withholding CNI probably due to an individually perceived risk of DGF by the transplant team. As such, the model does not necessarily give evidence of a protective effect of CNI against DGF and furthermore it is not intended for guiding immunosuppressive management. Importantly, a sensitivity analysis showed that excluding the CNI variable from the final model did not substantially impact its performance.

Fifth, it should be noted that the adjusted odds ratios of the predictors should not be interpreted as risk ratios or direct measures of risk reduction in the final prediction model. Rather they should be viewed as indicators that confirm the direction and relative strength of the associations between the predictors and DGF.

Sixth, model parameters such as serum creatinine and immediate urine output may have been biased by an early post-transplant dialysis procedure in children with DGF. In a dedicated sensitivity analysis, we excluded the 8 patients for whom the date of kidney transplantation and the date of the first post-transplant dialysis coincided. We then re-ran the model and excluded the CNI variable after forward selection. Both the discriminative performance (ROC-AUCs) and the direction of the associations of the selected predictors remained unchanged compared to the main model (Supplementary Tables 7,8), indicating that the inclusion of patients undergoing post-transplant dialysis on day 1 did not introduce relevant bias into the final model.

In conclusion, we developed a multivariable prediction model based on early (within the first 24 h after kidney transplantation) post-transplant parameters to assess the likelihood of DGF in pediatric kidney transplant recipients. This model can predict the occurrence of DGF in children with high accuracy, independent of pre-transplant risk factors. Thus, this model is a valuable tool for guiding patient-specific clinical decision-making post-transplant and it may facilitate future interventional trials of targeted pharmacological strategies against ischemia-reperfusion injury.

## Data Availability

The original contributions presented in the study are included in the article/Supplementary Material, further inquiries can be directed to the corresponding author.

## Footnotes

1
http://www.certain-registry.eu/

2
https://www.strobe-statement.org
