# Supplementary material for: Risk Assessment of Delayed Graft Function in Pediatric Kidney Transplantation – a CERTAIN Research Network Analysis
Source: Transpl Int. 2026 Mar 19;39:14640. doi: 10.3389/ti.2026.14640 (PMC13045783; doi:10.3389/ti.2026.14640)
Supplement: Supplementary file 1 [file Supplementaryfile1.docx]

**SUPPLEMENTARY MATERIAL**

**Contents:**

Description of the CERTAIN registry: completeness and quality of data

**Capsule Sentence**

Using CERTAIN registry data, we developed and validated the first pediatric post-transplant risk prediction model for delayed graft function (DGF). This model provides a tool for early risk stratification and clinical trial design for DGF in pediatric KTx.

**Description of the CERTAIN registry: data completeness and quality**

Data entry into the CERTAIN registry started in January 2011. As of November 2024, 96 centers from 26 countries are participating in the registry with 4172 patients enrolled. CERTAIN provides detailed data capture, thereby allowing an in-depth characterization of specific patient cohorts. Data are collected prior to kidney transplantation, at 1, 3, 6, and 12 months post-transplant and every 6 months thereafter. In addition, the CERTAIN dataset allows for detailed and flexible documentation of post-transplant follow-up through continuous entry of any number of relevant data (e.g., laboratory values, drug therapy). Specific case report forms (CRFs) can be developed to collect detailed and accurate information on relevant data and events of pediatric kidney transplantation in the peri- and post-transplant period. There are two data sets, the minimum required data set and the extended data set. The minimum required data set is mandatory for all participating centers. The extended data set provides deeper insight into the clinical course and treatment of patients by documenting additional items, some of which are predefined and some of which can be defined by the participating center.

The CERTAIN web application (accessible via http://www.certain-registry.eu/RegApp) has an automatic and manual data validation functionality. During data entry, the dataset is automatically validated against predefined plausibility ranges. In addition, the system has an integrated manual quality assurance process. First, documented data must be approved locally at the site; second, a data quality manager at the registry headquarters randomly checks data for plausibility. Only data that passes this quality assurance process are entered into the research database. These functions are available anytime, anywhere and require only a standard web browser and internet access.

| **Supplementary Table 1.** Parameters investigated in the combined pre-/post-transplant extended analysis | | | | |
| --- | --- | --- | --- | --- |
| **Parameters of interest** | | **DGF, N = 56** | **No DGF, n = 624** | ***P* Value** |
|  | |  |  |  |
| Dialysis mode before KTx, n (%) | |  |  | < 0.001 |
|  | Hemodialysis | 25 (45) | 180 (29) |  |
|  | Peritoneal dialysis | 29 (52) | 264 (42) |  |
|  | None | 2 (4) | 180 (29) |  |
| Type of donor, n (%) | |  |  | < 0.001 |
|  | Deceased donor after cardiac death | 2 (4) | 10 (2) |  |
|  | Deceased donor after brain death | 41 (73) | 299 (48) |  |
|  | Unknown | 9 (16) | 97 (16) |  |
|  | Living donor | 4 (7) | 218 (35) |  |
| Cold ischemia time [min], mean ± SD | | 831 ± 365 | 609 ± 421 | < 0.001 |
| Age of recipient [y], mean ± SD | | 11 ± 5.8 | 9.8 ± 5.3 | 0.151 |
| Donor hemodynamic instability, n (%) | |  |  | < 0.001 |
|  | No/unknown | 16 (29) | 387 (62) |  |
|  | Yes | 40 (71) | 237 (38) |  |
| HLA-DR mismatches, n (%) | |  |  | 0.053 |
|  | 0 | 19 (34) | 169 (27) |  |
|  | 1 | 36 (64) | 379 (61) |  |
|  | 2 | 1 (2) | 76 (12) |  |
| Donor sex, n (%) | |  |  | 0.265 |
|  | Female | 28 (50) | 264 (42) |  |
|  | Male | 28 (50) | 360 (58) |  |
| Surgical complications post-transplant | |  |  | < 0.001 |
|  | No | 49 (89) | 605 (99) |  |
|  | Yes | 6 (11) | 7 (1) |  |
| Rate of change in recipient’s serum creatinine, mean ± SD | | -0.006 ± 0.023 | -0.064 ± 0.092 | <0.001 |
| Immediate urine production, n (%) | |  |  | <0.001 |
|  | No | 25 (68) | 64 (17) |  |
|  | Yes | 12 (32) | 304 (83) |  |
| Calcineurin inhibitor therapy, n (%) | |  |  | 0.260 |
|  | No | 3 (6) | 16 (3) |  |
|  | Yes | 47 (94) | 512 (97) |  |
| For binary and categorical variables, numbers and percentages are given. For continuous variables, mean and standard deviation are presented.  For metric variables a two-sided t-test was calculated. To compare non-metric variables the Chi-squared test was applied. For all tests p<0.05 was considered significant.    *HLA, human leukocyte antigen.* | | | | |

| **Supplementary Table 2**. Types of graft ureteral stent | | | | |
| --- | --- | --- | --- | --- |
| **Parameters of interest** | | **DGF, n = 59** | **No DGF, n = 635** |  |
|  | |  |  |  |
| Stenting of the graft ureter by catheter type, n (%) | |  |  |  |
|  | Cystofix catheter | 2 (4) | 5 (1) |  |
|  | Single J catheter | 28 (52) | 206 (34) |  |
|  | Double J catheter | 12 (22) | 175 (29) |  |
|  | Splint, not specified | 3 (6) | 121 (20) |  |
|  | None | 9 (17) | 103 (17) |  |
|  | Missings | 5 | 25 |  |
| For categorical variables, numbers and percentages are given. Chi-squared test was applied to compare non-metric variables. For all tests p<0.05 was considered significant. | | | | |

| **Supplementary Table 3:** Timepoint of initiation of post-transplant dialysis within the first seven days after transplant surgery in the DGF cohort. | | | | | | | |
| --- | --- | --- | --- | --- | --- | --- | --- |
|  | **Day 1** | **Day 2** | **Day 3** | **Day 4** | **Day 5** | **Day 6** | **Day 7** |
| Number of patients | 8 | 16 | 18 | 4 | 3 | 2 | 5 |
| In 3 patients the timepoint of initiation of post-transplant dialysis within the first seven days after transplant surgery was not documented. | | | | | | | |

| **Supplementary Table 4:** Parameters chosen for the combined pre- and post-transplant DGF risk prediction model by forward selection | | | | |
| --- | --- | --- | --- | --- |
| **Parameters** | | **Regression Coefficient** | **Odds Ratio**  **[lower – upper CI]** | **P Value** |
|  | |  |  |  |
| Intercept | | 2.066 | 7.893 [1.422 – 39.433] | **0.020** |
|  | |  |  |  |
| Surgical complications post-transplant | |  |  |  |
|  | No | *Reference* | - | - |
|  | Yes | 1.754 | 15.2 [4.35 – 55.3] | 0.020 |
|  |  |  |  |  |
| Immediate graft urine production | |  |  |  |
|  | No | *Reference* | - | - |
|  | Yes | -2.264 | 0.104 [0.043 – 0.239] | <0.001 |
|  |  |  |  |  |
| Number of HLA-DR mismatches | |  |  |  |
|  | 0 | *Reference* | - | - |
|  | 1 | 0.515 | 1.673 [0.699 – 4.332] | 0.254 |
|  | 2 | -1.509 | 0.221 [0.002 – 2.153] | 0.231 |
| Calcineurin inhibitor as part of post-transplant immunosuppressive therapy | |  |  |  |
|  | No | *Reference* | - | - |
|  | Yes | -2.787 | 0.062 [0.014 – 0.300] | 0.001 |
|  |  |  |  |  |
| Rate of change in recipient’s serum creatinine | | 0.240 | 1.272 [1.123 – 1.466] | <0.001 |
| The reference category describes the variable characteristic to which the risk calculation of the other parameters refers. The reference category was randomly selected.  *HLA, human leukocyte antigen.* | | | | |

| **Supplementary Table 5.** Parameters selected for the DGF risk assessment model with exclusion of the CNI parameter after forward selection | | | | |
| --- | --- | --- | --- | --- |
|  | | **Regression Coefficient** | **Odds Ratio**  **[95% CI]** | ***p* value** |
|  | |  |  |  |
| *Intercept* | | *-0.652* | 0.520 [0.317 – 0.841] | <0.001 |
|  | |  |  |  |
| Surgical complications post-transplant | |  |  |  |
|  | No | *Reference* | - | - |
|  | Yes | 1.574 | 4.827 [1.147 – 21.35] | <0.001 |
|  |  |  |  |  |
| Immediate urine production | |  |  |  |
|  | No | *Reference* | - | - |
|  | Yes | -2.049 | 0.128 [0.055 – 0.277] | <0.001 |
|  |  |  |  |  |
| Rate of change in recipient’s serum creatinine | | 0.233 | 1.263 [1.112 – 1.462] | <0.001 |
| The reference category describes the variable characteristic to which the risk calculation of the other parameters refers. The reference category was randomly selected. | | | | |

| **Supplementary Table 6.** ROC-AUCs of the DGF risk assessment model with exclusion of the CNI parameter after forward selection in different patient cohorts | | |
| --- | --- | --- |
| **Type of cohort** | **ROC-AUC** | |
|  | |  |
| ROC-AUC on training cohort | | 0.8819 |
| ROC-AUC on validation cohort with complete cases | | 0.8905 |
| Mean ROC-AUC of imputation validation datasets | | 0.8848 |
| ROC-AUC on merged imputed validation cohort | | 0.9171 |
| ROC-AUC, area under the receiver operating characteristic curve.  There were no missing data in the selected predictors. | | |

| **Supplementary Table 7.** Parameters selected for the DGF risk assessment model with exclusion of the CNI parameter after forward selection performed on data excluding patients with post-transplant dialysis on the same day as KTx (n=686 patients) | | | | |
| --- | --- | --- | --- | --- |
|  | | **Regression Coefficient** | **Odds Ratio**  **[95% CI]** | ***p* value** |
|  | |  |  |  |
| *Intercept* | | *-0.788* | 0.454 [0.269 – 0.751] | <0.001 |
|  | |  |  |  |
| Surgical complications post-transplant | |  |  |  |
|  | No | *Reference* | - | - |
|  | Yes | 1.795 | 6.022 [1.372 – 28.68] | <0.001 |
|  |  |  |  |  |
| Immediate urine production | |  |  |  |
|  | No | *Reference* | - | - |
|  | Yes | -2.346 | 0.095 [0.035 – 0.227] | <0.001 |
|  |  |  |  |  |
| Rate of change in recipient’s serum creatinine | | 0.219 | 1.245 [1.088 – 1.456] | <0.001 |
| The reference category describes the variable characteristic to which the risk calculation of the other parameters refers. The reference category was randomly selected. | | | | |

| **Supplementary Table 8.** ROC-AUCs of the DGF risk assessment model with exclusion of the CNI parameter after forward selection performed on data excluding patients with post-transplant dialysis on the same day as KTx (n=686 patients) | | |
| --- | --- | --- |
| **Type of cohort** | **ROC-AUC** | |
|  | |  |
| ROC-AUC on training cohort | | 0.8954 |
| ROC-AUC on validation cohort with complete cases | | 0.8876 |
| Mean ROC-AUC of imputation validation datasets | | 0.8858 |
| ROC-AUC on merged imputed validation cohort | | 0.9192 |
| ROC-AUC, area under the receiver operating characteristic curve.  There were no missing data in the selected predictors. | | |

**Supplementary Figure 1:** Study flow-chart for the extended analysis of combined pre- and post-transplant risk factors for DGF


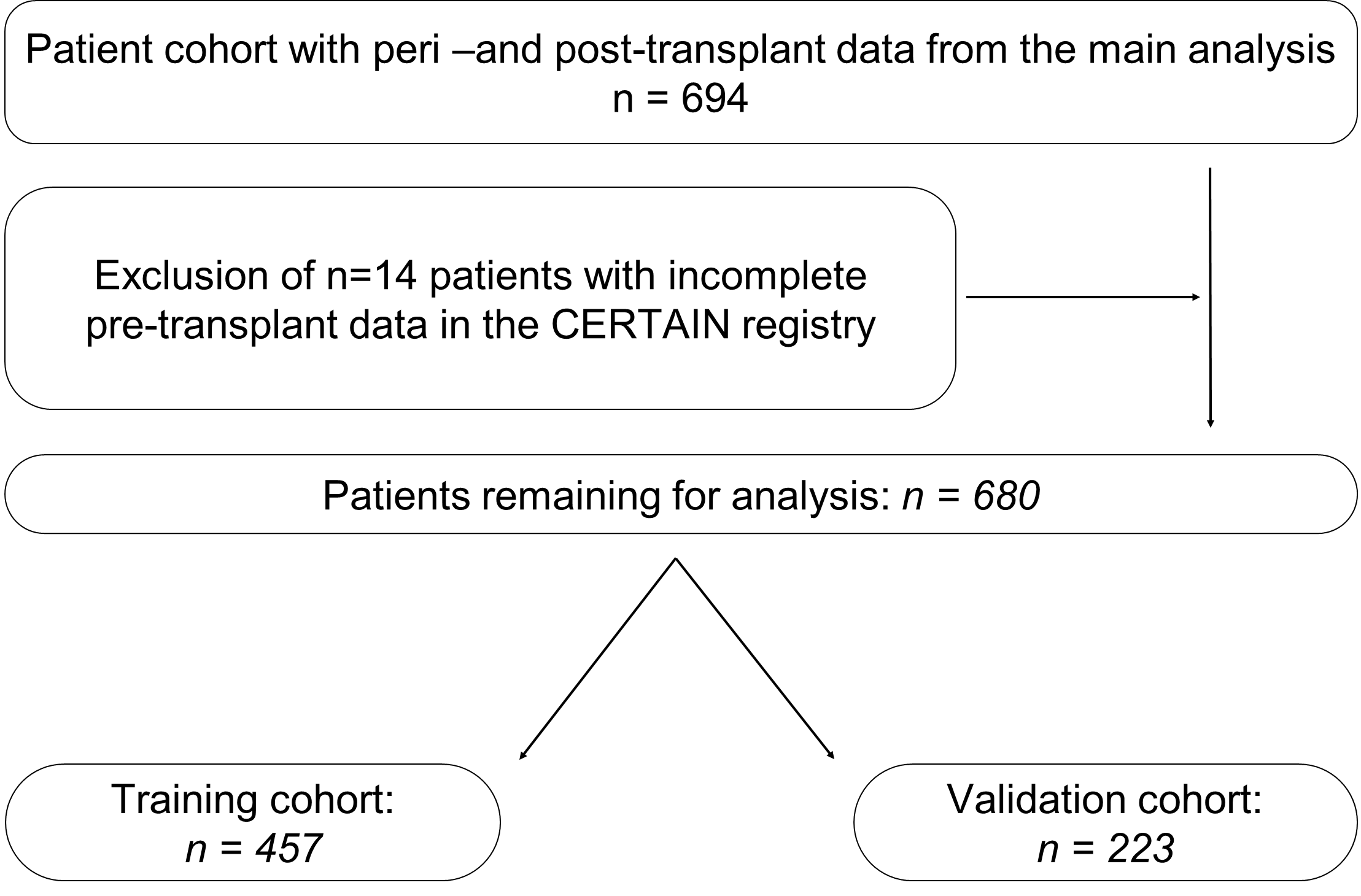


**Figure Legend:** The extended analysis of combined pre- and post-transplant factors for predicting DGF in pediatric kidney transplantation was performed in a subcohort of the main study cohort. To generate this subcohort, we had to exclude n=14 patients from the main study cohort because these patients lacked complete pre-transplant data entries. This extended analysis included all post-transplant parameters for DGF prediction as selected by forward selection during the main analysis. In addition, we integrated pre-transplant data derived from the CERTAIN registry for these patients. The patients remaining for this extended analysis were again divided 2:1 into a training cohort and a validation cohort.

**Supplementary Figure 2:** Calibration plot of the combined pre- and post-transplant pediatric DGF risk prediction model


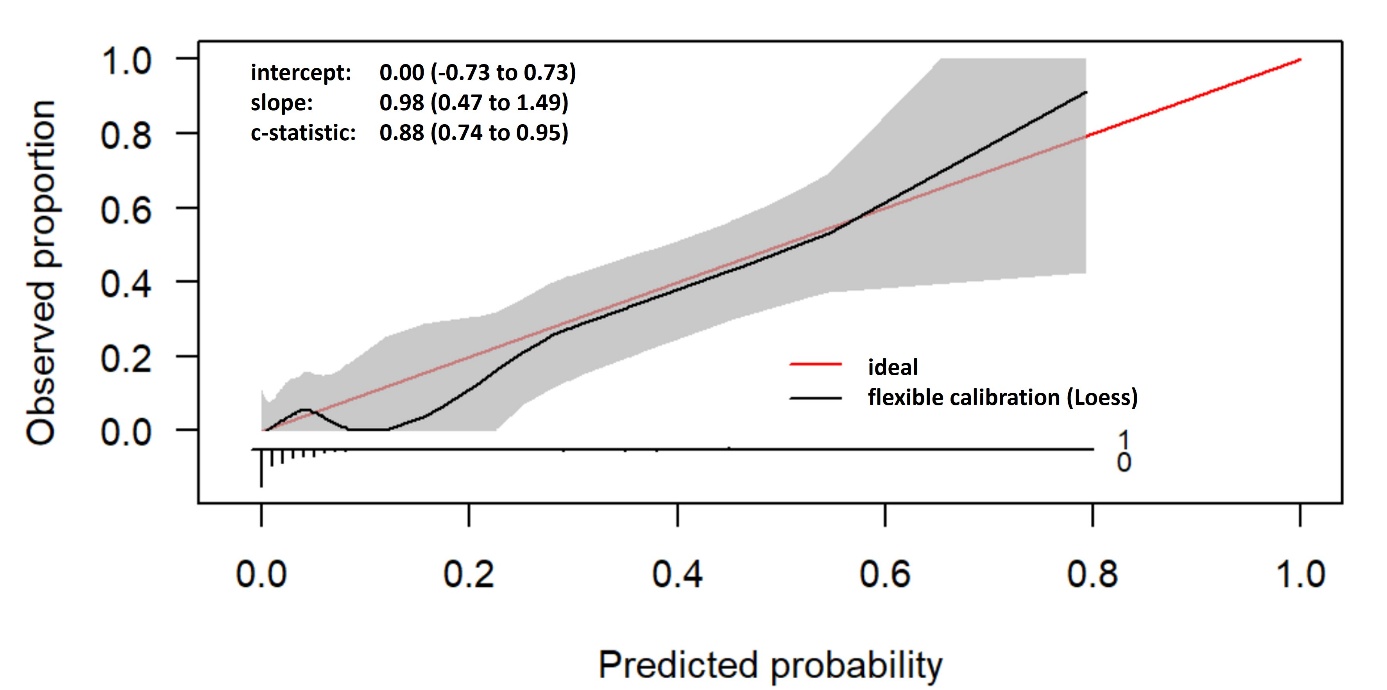


**Figure Legend:** Figure 2 shows the calibration plot of the pediatric DGF risk assessment model.
